# Voluntariness and type of digital device usage: A study in terms of Vygotsky's cultural–historical perspective

**DOI:** 10.3389/fpsyg.2023.1111613

**Published:** 2023-03-06

**Authors:** Arina Shatskaya, Margarita Gavrilova, Elena Chichinina

**Affiliations:** ^1^Laboratory of Childhood Psychology and Digital Socialization, Psychological Institute of Russian Academy of Education, Moscow, Russia; ^2^Department of Education Psychology and Pedagogy, Faculty of Psychology, Lomonosov Moscow State University, Moscow, Russia

**Keywords:** preschool children, gadgets, self-regulation, working memory, inhibitory control, cognitive flexibility, executive functions

## Abstract

**Introduction:**

In recent years, exposure to digital devices during the development stages of a child has been steadily increasing. Exploring the relationship between children's digital device exposure and their voluntariness still shows quite contradictory results. Screen time is the most studied factor on this issue. The purpose of the present study was to suggest the type of digital device used is another factor in addition to screen time. In accordance with the research hypothesis, the use of digital devices as a psychological means is related to higher voluntariness scores.

**Methods:**

The study sample was recruited from Moscow kindergartens and schools: preschoolers aged 5–6 years (*n* = 408) and 6–7 years (*n* = 351) and schoolchildren aged 7–8 years (*n* = 253) and 9–10 years (*n* = 101). The study participants took part in a voluntariness assessment performed *via* executive functions' (EF) evaluation as well as in a semi-structured interview to identify the type and frequency of digital device usage.

**Results:**

There are three findings of the present study, which are given as follows. First, the “frequency of digital device usage” predictor proved its statistical significance for verbal working memory, inhibition, and cognitive flexibility at 7–8 years, and for verbal working memory at 6–7 years. Second, the number of children who use digital devices as a psychological means increases as they grow older. Third, the number of children who use digital devices as a psychological means tends to demonstrate higher mean scores for any executive function skills at 6–7 and 9–10 years and for visual and verbal working memory at 7–8 years. Multiple regression models did not confirm the significance of the “type of digital devices usage” predictor for executive function skills considering the participants' individual characteristics.

**Discussion:**

“Type of digital devices usage” predictor is assumed to be more applicable to children at the end of primary school and older when exploring executive function skills in the context of children's digital device exposure.

## 1. Introduction

Currently, there is an increasing digitalization of human life. The involvement of people around the world in the digital environment is growing annually. This trend is seen in all age groups, including the preschool community (Sowmya and Manjuvani, 2019; Konca, 2022; Scott, 2022). For instance, by 2022, 60% of the US parents reported that their child had started using a smartphone at the age of 5 years, while 31% of them explained that their children had had digital experiences even before they were 2 years old (DataReportal, 2020). In addition, as the age at which children have their first digital experience on a device (mobile phones, tablets, smart watches, etc.) becomes lower, so does the frequency of digital usage (Smirnova et al., 2022). For example, the total screen time of preschoolers and primary school children varies from 1 to 3 h per day (Soldatova and Vishneva, 2019; Konca, 2022). It is also worth mentioning that the use of digital Internet technologies has grown not only in the northern hemisphere where most of the countries are wealthy but also in the southern hemisphere (Byrne et al., 2016). Thus, the trend toward earlier familiarization of children with digital devices (herein after—DD) and toward an increasing frequency of interaction with such devices has, in fact, become global.

Currently, both adults and children acquire a significant part of social experience *via* interaction with DD–digital socialization (Soldatova and Vojskunsky, 2021; Veraksa A. N. et al., 2021; Podosokorsky, 2022; Sysoeva and Yaroshevskaya, 2022; Zhuravlev et al., 2022). A gradual digital transformation of the educational process in schools (Koinova-Zollner et al., 2022) and preschool institutions (Komarova, 2022) is also taking place. The phenomenon as described creates a new type of child development, a “digital childhood” (Rubtsova, 2019a,b). Interaction with DD at an early age frequently begins on the grounds of entertainment: watching cartoons and playing games (Rideout and Robb, 2017; Nikitina and Rytova, 2019). Later, the content becomes more variable, and new functions, such as communication, searching for information, doing homework, etc., are added (Veraksa et al., 2022).

The intense integration of digital technologies into the children's day-to-day activities from the earliest age poses the question of the potential impact on the children's development. Due to its uncertainty and ambiguity, the DD influence on child development is a matter of debate and discussion in the scientific community. On the one hand, a whole range of research works revealed that some computer games develop planning skills and logical thinking in children (Batenova, 2011), as well as visuomotor coordination (Alekhin and Pul'cina, 2020), spatial orientation (Subrahmanyam and Greenfield, 1994), and visual working memory (Blacker et al., 2014). In addition, it has been shown that digital educational software also positively affects children's cognitive development (Kotler et al., 2016; Klopotova and Romanova, 2020).

On the other hand, there are multiple research data confirming the negative DD influence on child development. For instance, watching too much video content and frequent Internet usage can result in vocabulary deficiencies and lower language skills (Nirwana et al., 2018; Takeuchi et al., 2018; Nikitina and Rytova, 2019; Sari, 2020) and inhibit the processes of emotional development and child socialization because of the lack of two-way communication (Suhana, 2017; Sapardi, 2018). Moreover, a negative effect, especially for attention focus, was registered for cognitive development processes (Christakis et al., 2004; Haier et al., 2009; Cho and Lee, 2017).

Thus, clarifying the issue will help us understand the peculiarities of teaching children in the digital era, which makes the topic particularly timely (Nechaev and Durneva, 2016).

What factor can adequately represent the effect of DD usage on children's mental development and future performance? Screen time is the most widely studied factor of how gadgets impact a child's development and voluntariness (Jusiene et al., 2020; Martins et al., 2020; McHarg et al., 2020; Vohr et al., 2021). However, not all studies support the predictive power of screen time (Huber et al., 2016; Radesky and Christakis, 2016; Jusiene et al., 2020; Papadakis et al., 2022). What other factors can be explored besides screen time? The present study attempts to answer this question based on the cultural–historical perspective. The advantage of this approach lies in the fact that it considers human activity as the product of historical social development as a result of the interaction of an individual with the objects in the environment, which also includes DD in the present world (Soldatova and Vojskunsky, 2021).

According to Vygotsky, the developmental act in its essence consists of achieving the mastery of cultural means (signs, sign systems, certain ways of activity, etc.) and leads to the transition from non-mediated to mediated actions. Vygotsky's cultural–historical perspective describes three stages of child cultural development. In the first stage, children do not master any cultural experience or signs, but the adults do it for them. In the second stage, a child moves to master the signs by using them in his/her own interaction with others, as if controlling their behavior. For example, a toddler points at a toy and the adult names it and gives it to the toddler. Finally, in the third stage, children learn to apply signs to their own behavior (Vygotsky, 1983). Thus, the development of voluntariness turns out to be an important indicator of a child's mental development (Solovieva et al., 2021), and therefore, it has gained utmost research interest.

If we talk about voluntariness within the framework of the cultural–historical perspective, it is necessary to distinguish between voluntary and volitional behavior (voluntariness and will). A key feature of voluntary behavior is the awareness of the ways of one's or own actions, as well as the ability to mediate them using cultural signs (Smirnova, 2015). Voluntariness is directed at oneself, at the means and methods of one's external or internal activity. Will is a motivation that encourages a person to take some action (Smirnova, 2015). Thus, these two concepts have not only much in common but are also diverse. On the one hand, if the motive and purpose of the action do not belong to the child himself/herself (e.g., acting according to instructions), the child's actions may be voluntary and mediated but not volitional. On the other hand, a strong-willed person with a stable system of motives, even having a definite goal, may not have the voluntariness of his/her actions. The development of will can be conceived as the formation of stable motivation and the formation of the child's own stable desires and aspirations. From this point of view, the development of voluntariness can be considered as mastering the means of organizing one's behavior, which allows the person to objectify and realize his/her own actions. A person with developed voluntariness demonstrates organized and consciously controlled behavior, which he/she can subordinate to norms and rules.

The present study deals with the development of voluntariness from the perspective of executive functions (EF) within Miyake's approach (Miyake et al., 2000). According to this cognitive concept, EF are a family of top-down mental processes required for voluntary planning, control, and regulation of one's actions in accordance with the current task and selective attention to stimuli (Diamond, 2013). Fundamental executive function skills are working memory (visual and verbal) that preserves and manipulates information that is not available for perception anymore; inhibitory control that inhibits domineering responses in favor of the one required by the task; and cognitive flexibility (switching between several alternatives, rules, or perspectives) (Miyake et al., 2000). A large range of research works proved that this three-component model is enough to describe the features of EF structure in children (Diamond and Lee, 2011; Almazova et al., 2016). Executive functions are often considered the most reliable predictors of success in adulthood to a higher degree than even the IQ level or the socioeconomic status (Moffitt et al., 2011). All these arguments elaborate on the attention paid to appropriating EF (voluntariness) as a key developmental task in preschool childhood (Almazova et al., 2016) and its relation to contemporary cultural practices, especially those including DD usage.

However, understandings of the voluntariness concept within the cultural–historical perspective and the cognitive perspective are different. In the cultural–historical perspective, Venger's theory of abilities and his diagnostic toolkits are widely used when exploring voluntariness (Venger, 1986). The theory of abilities is based on the idea that voluntariness develops in relation to cultural means (sensory standards, visual models, etc.). The cognitive approach evaluates the development of voluntariness through the concept of EF as a set of cognitive skills. However, Almazova et al. (2016) assessed child voluntariness using both the Venger toolkit and the EF diagnostics and compared the results. The same reality is studied in each of the approaches, and voluntariness can be studied using executive function diagnostics.

Vygotsky considered sign usage as a key moment in the development of higher mental functions (Vygotsky, 1983). It is important to distinguish between the concept of a practical means and the concept of a sign. A human being uses both psychological and practical means in his/her activity (Cole and Engeström, 1993; Vygotsky, 2006; Nussbaumer, 2012). The former includes signs that transform the mental processes of a person and allow mastering one's own behavior and influencing other's behavior. Since psychological means are directed inwardly, they allow the regulation of one's own and other people's behavior, while practical means are directed outwardly and are meant for the transformation of external objects (Vygotsky, 1982). All these means have natural and cultural components, that is, a material aspect with certain physical properties and a social one reflecting culturally established ways of their use (Veresov and Veraksa, 2022).

The Internet and DD usage can be considered as a new means of symbolic mediation of activity. All types of activities with DD can perform both as practical and as psychological means simultaneously (Rubtsova, 2019a,b; Veraksa N. E. et al., 2021). Using DD, children can directly affect the objects around them to fulfill their needs (a DD acts as a practical means). At the same time, DD can be used to influence one's own and other people's behavior, and in this study, the “sign” aspect of a DD manifests itself (e.g., the alarm, notes, or calendar functions of a smartphone serve as a means of self-regulation). In addition to DD usage as a psychological means, DDs are used for reading, playing gadget games, etc., which changes the structure of the related psychological processes compared to their natural counterparts—reading books and playing non-virtual games (Vygotsky, 1982; Rubtsova, 2019a,b). Thus, by what criterion can the varieties of DD usage be distinguished from each other?

According to the cultural–historical activity theory, activity is a holistic unit of analysis directed by a group of individual goals and motives (Leontiev, 1974; Galperin, 1992; Davydov, 1999). Within this concept, any activity has a three-component structure: activity–action–operation. Activity is determined by a motive, action is determined by a goal, and operations are determined by the specific conditions of the process (Leontiev, 1978).

From this point of view, the DD usage can be considered in two ways. (A) On the one hand, the motive of activity involving a DD is to actively transform and regulate the behavior and mental processes of oneself or others. For example, a child makes a note in the calendar application so as not to forget to complete a project (a gadget as a means of memory regulation) or a child communicates with relatives using WhatsApp (a gadget as a means of communication to influence interlocutors). Using DD as a means, people aim to get a certain outcome in regulating their own or someone's else behavior. In this case, we talk about the usage of DD as an active psychological means.

(B) On the other hand, a DD can be used directly for interaction with another DD to enjoy this process. For example, some people enjoy surfing the Internet without any purpose, and many play digital games or watch video content. Since people are enjoying the process, it matters more than the result. In this case, we believe that DDs are used as entertainment (Veraksa and Buhalenkova, 2017).

Thus, it seems interesting to consider whether DD usage as a psychological means contributes to a higher degree of voluntariness. We do not deny that a child can simultaneously develop and change the behavior and mental functions of himself/herself and others even in the case of using DD for entertainment. However, we suggest that, in these cases, the self-regulating act is not the main purpose of the child's activity.

The main purpose of this study was to suggest the type of digital device usage as another factor in addition to screen time when exploring the relationship of voluntariness with DD usage. It was hypothesized that DD usage as a psychological means is related to higher executive functioning scores.

## 2. Materials and methods

### 2.1. Participants

The participants were recruited from several public kindergartens and primary schools in Moscow. The present study was performed with the data acquired from the four age groups: (a) *preschoolers aged 5–6 years* from the senior kindergarten groups (*n* = 408, *M* = 64 months, *SD* = 4; 47% boys), (b) *preschoolers aged 6–7 years* from the preschool kindergarten groups (*n* = 351, *M* = 64 months, *SD* = 3.8; 49.5% boys), (c) *schoolchildren aged 7–8 years* from the first grade (*n* = 253, *M* = 90 months, *SD* = 6.2; 39.5% boys), and (d) *schoolchildren aged 9–10 years old* from the third grade (*n* = 101, *M* = 118 months, *SD* = 3.7; 49.5% boys).

Participation in the study for preschoolers and schoolchildren was organized on a voluntary non-reimbursable basis. The parents of each participant signed the informed consent for their child's participation. All the participants were involved in basic education programs and had no developmental delays or disabilities. All the assessment tasks were performed during an individual meeting with each participant lasting 30–35 min in a quiet room of the child's kindergarten or school in the first half of the day.

### 2.2. Measures

#### 2.2.1. Voluntariness

The level of voluntariness development was assessed by evaluating the EF skills: visual and verbal working memory, inhibitory control, and cognitive flexibility.

Visual working memory was assessed *via* the “*Memory for design*” subtest (Korkman et al., 2007). Children could obtain (1) “Content scores”—for remembering the image elements correctly, (2) “Spatial scores”—for indicating the correct location of the image elements in the field, (3) “Bonus scores”—for meeting both criteria, and (4) “Total score” as the sum of all three parameters (150 points max.).

The “*Sentence Repetition*” subtest (Korkman et al., 2007) was used to assess verbal working memory. This technique consists of 17 sentences that gradually become longer and more complex grammatically. Each sentence recalled accurately is awarded 2 points. In case of three or more errors, or no answer at all, 0 points are assigned. If the respondent receives 0 points four times consecutively, the trial stops (34 points max.).

Inhibitory control was evaluated by means of the “*Inhibition*” subtest (Korkman et al., 2007). This technique consists of two blocks: a row of black and white circles and squares, and a row of black and white arrows pointing upward and downward. With both blocks, children had to perform two tasks: (1) “Naming”—to name all figures as quickly as they possibly could and (2) “Inhibition”—to name the opposite figure or direction as quickly as they possibly could (a square instead of a circle and vice versa). The number of corrected and not corrected errors and the time spent on the task were registered. These parameters were converted into a combined score for convenience in accordance with the authors' guideline (20 points max.).

We assessed cognitive flexibility in preschoolers and schoolchildren by different age-appropriate techniques. Primary school children were asked to perform the third task from the aforementioned “Inhibition” subtest, the “Switching” (Korkman et al., 2007). It required naming the figures in a row as quickly as possible according to the following set of rules: if a figure was white, it should be named oppositely (it was correct to say “a circle” instead of “a square,” and vice versa), but if a figure was black, it should be named correctly (a circle was a circle). The number of corrected and not corrected errors and the time spent on the task were registered.

In preschoolers, cognitive flexibility was assessed by the “*DCCS*” tool consisting of three tasks (Zelazo, 2006). In the first one, children had to sort the cards by color; in the second one, children had to sort the cards by their shape, and in the third one, children had to sort the cards following a special rule (if the card had a black frame, it was to be sorted by color; if it did not have any, it was to be sorted by shape). Each correct sorting was awarded 1 point (24 points max.).

The validity of using the psychometric tests to evaluate EF skills is supported by the fact that these tests have already been approved by the standardized diagnostic system to be used when it is necessary to acquire relevant measurements for all groups under study (Veraksa et al., 2020). At the same time, previous studies proved the diagnostic tools of the standardized NEPSY-II and DCCS tests to be analogous to the Venger tests (Venger and Kholmovskaya, 1978), which are widely used within the cultural–historical concept to assess voluntariness (Almazova et al., 2016).

#### 2.2.2. DD usage

To assess the type of interaction with digital devices, a semi-structured interview was elaborated. The interview included two blocks of questions about the frequency of DD usage and the ways by which children use them. The first block concerned the type of usual activity with digital devices:

(Q1) How often do you use gadgets for communication (conversations and messaging)?
(Q2) How often do you use gadgets for information search?
(Q3) How often do you use gadgets to do homework?
(Q4) How often do you use gadgets for the purpose of self-organization (alarm clock, maps, planner, etc.).
(Q5) How often do you use gadgets to take pictures and make videos?
(Q6) How often do you use gadgets to listen to music?
(Q7) How often do you use gadgets to draw?
(Q8) How often do you use gadgets to play games?
(Q9) How often do you use gadgets to watch cartoons, videos, and movies?

The responses received were recorded and classified into four categories: hardly ever, sometimes, often, and very often. Then, the children's responses were divided into three categories: (1) DDs are mostly used as “psychological means” (most frequently used categories are a–g); (2) “for entertainment” (h, i); and (3) “both” (a–i).

The second block of questions related to the frequency of using gadgets. The participants answered the question:

(Q10) How often do you use your smartphone/tablet/laptop/computer? The responses received fell into one of the three categories: rarely—several times a week or less; every day; very often—more than 2–3 h every day.

The age, gender, and non-verbal fluid intelligence and parental educational level were included as participants' characteristics. The child's non-verbal fluid intelligence was assessed with the help of Raven's Colored Progressive Matrices (Raven, 1998). The participants performed assessment tasks until four mistakes were made and the number of tasks completed correctly was counted. Parental educational level was assessed in the parental interview and attributed to one of the following categories: secondary general education; specialized secondary education; incomplete higher education; higher education; and academic degree.

The research hypotheses were tested using multiple linear regression models and *t*-tests. All computations were processed using the R language software (version 4.1.2) and Jamovi (version 2.3.16). G^*^Power 3.1.9.4 was used to perform the *a posteriori* power analysis.

## 3. Results

At the first stage of analysis, the general descriptive data on DD usage and EF skills were acquired for all the age groups from the youngest one [5–6 years old (years old) to the oldest (9–10 years old (y.o.)]. The acquired data are presented in the following paragraph.

### 3.1. DD usage

The descriptive data on the features of DD usage are summarized in Table 1. The percentage of children who do not use DD tends to decrease as children grow older. However, in the senior kindergarten group, 67.9% of the children use gadgets, and in the first grade, nearly all of them use DD (99.15%). By the third grade, all children use digital devices (100%). At the same time, the number of children who use DD very often, that is, for more than 2–3 h daily, grows from the senior kindergarten to the third grade (chi-square test, χ^2^ = 271, *df* = 15, *p* < 0.000).

**Table 1:** Characteristics of child digital device usage at different ages.

**Age period**	**Total no DD usage (%)**	**Total DD usage (%)**	**DD usage**			
			**Type of DD usage (%)**		**Frequency of DD usage (%)**	
5–6 y. o.	32.1	67.9	Mean	7.4	Rarely	8
			Entertainment	35.2	Everyday	48.7
			Both	25.3	Very often	8
6–7 y. o.	5.4	94.6	Mean	10.5	Rarely	41.7
			Entertainment	60.2	Everyday	47.4
			Both	23.8	Very often	6.0
7–8 y. o.	0.008	99.15	Mean	20	Rarely	0
			Entertainment	78.0	Everyday	64.4
			Both	2	Very often	35.6
9–10 y. o.	0	100	Mean	38.0	Rarely	0
			Entertainment	55.7	Everyday	43.0
			Both	6.3	Very often	57.0

We can state that most children use DD as entertainment in all four age groups. The percentage of children who use DD as a psychological means increases from 7.4% for the 5–6-year-olds (the senior group) to 38% for the 9–10-year-olds (the third grade) (χ^2^ = 117, *df* = 6, *p* < 0.000). Thus, the older the children, the more likely they will use DD mainly as a psychological means.

### 3.2. EF skills and type of DD usage

The descriptive statistics for all EF skills for all age groups, delineated by the type of DD usage, are presented in Table 1. In this study, we focused on two types of DD usage—as a means or for entertainment—and the children were attributed to one of the two groups according to their preferred type of usage. The differences in EF skills between these two groups were analyzed using Student's *t*-test/Mann–Whitney U-test for normally/non-normally distributed interval variables (see Table 1).

As a result, no significant differences were found between the “means” and “entertainment” groups for the 5–6- and 6–7-year-old children. As regards the 7–8-year-old children and their EF skills, a significant difference was found for verbal working memory in favor of the “means” group (Rank-Biserial Correlation as an effect size = 0.184). For the 9–10-year-old children, significant differences between the two groups were found for verbal working memory (the Cohen's *d* as an effect size = 0.504) and for inhibition (Cohen's *d* = 0.487) in favor of the “means” one.

We decided to evaluate the general EF skills trend for all groups under study. As can be seen from Table 2, for the children 5–6 years old, averaged EF skills in the “means” group never exceed the values of the “entertainment” group. Among the 6–7-year-old children, the “means” group exceeds the “entertainment” group in average values for all EF skills. Among the 7–8 year-old children, the “means” group exceeds the “entertainment” group in average values for visual and verbal working memory. Among the 9–10-year-old children, the “means” group exceeds the “entertainment” group in average values for all EF skills (in view of equal mean values for visual working memory, the medians were estimated: for the “means” = 134 and for the “entertainment” = 129).

**Table 2:** Descriptive data for EF development in children from 5 to 10 years.

**Age period**	**Type of DD usage**	**Visual working memory**	**Verbal working memory**	**Inhibition**	**Cognitive flexibility**
5–6 y. o.	Mean	67.2 (21.9)	16.9 (6.64)	9.0 (0.01)	7.5 (3.19)
	Entertainment	79.4 (20.3)	19.1 (5.04)	9.09 (0.29)	7.54 (3.14)
	Both	72.3 (19.9)	18.8 (4.66)	9.18 (0.385)	8.29 (2.48)
	*Mean vs. Entertainment*	*T* = 1.862, *p* = 0.067	*T* = 1.312, *p* = 0.194	*U* = 300, *p* = 0.289	*U* = 328, *p* = 0.829
6–7 y. o.	Mean	88.6 (23.8)	22.1 (3.99)	11.2 (3.58)	9.24 (2.41)
	Entertainment	82.9 (24.0)	21.2 (4.89)	11.0 (3.05)	8.87 (2.58)
	Both	84.2 (21.8)	21.6 (4.60)	10.8 (3.09)	9.43 (2.53)
	*Mean vs. Entertainment*	*U* = 2888, *p* = 0.200	*T* = −0.935, *p* = 0.351	*U* = 3183, *p* = 0.653	*U* = 3190, *p* = 0.623
7–8 y. o.	Mean	113 (28.1)	23.0 (4.25)	11.5 (2.85)	10.6 (2.96)
	Entertainment	106 (31.4)	21.8 (3.94)	12.0 (3.11)	11.2 (3.40)
	Both	110 (24.7)	23.0 (5.83)	12.8 (3.03)	8.20 (0.84)
	*Mean vs. Entertainment*	*T* = −1.407, *p* = 0.161	***U*** **=** **3799**, ***p*** **=** **0.047**	*U* = 3721, *p* = 0.174	*U* = 3935, *p* = 0.405
9–10 y. o.	Mean	125 (19.9)	27.2 (5.07)	15.3 (2.58)	14.0 (3.32)
	Entertainment	125 (20.0)	25.0 (3.86)	13.9 (3.28)	13.3 (3.91)
	Both	130 (25.5)	25.4 (4.39)	16.6 (2.19)	16.2 (1.92)
	*Mean vs. Entertainment*	*T* = 0.055, *p* = 0.956	***T*** **=** **−2.13**, ***p*** **=** **0.037**	***T*** **=** **−2.06**, ***p*** **=** **0.043**	*T* = −0.80, *p* = 0.430

Standard deviation (SD) is reported in parentheses. **Bold** text indicates a statistically significant predictor (*p*-value < 0.05).

*A posteriori* power analysis was performed for all the comparisons between the “means” and “entertainment” groups for all test groups considering the achieved effect sizes in each comparison, the size of the groups, and α = 0.05 (total 16 power values). As a result, the maximum statistical power among all comparisons was 68%. We assumed the unbalanced size of groups to be the reason for such a low statistical power, mainly the small size of the “means” group. Low statistical power combined with the fact that, in 62.5% (10 out of 16) of comparisons, there was a tendency for the “means” group to prevail over the “entertainment” group, suggests that this negative result may be false (type II error).

#### 3.2.1. Multiple linear regressions for EF skills

At the second stage of analysis, multiple linear regression models were built to assess the contribution of the DD usage type to the EF skills for all test groups, considering the participants' individual characteristics. Thus, 16 linear regression models (4 EF skills × 4 test groups) were simulated. The dependent variables were EF skills (visual and verbal working memory, inhibition, and cognitive flexibility), as measured in each test group. The following variables were used as predictors in the models: (a) the type of DD usage—mainly as entertainment; as both entertainment and means; as a psychological means (hereinafter referred to as a “type-predictor”) and (b) the frequency of DD usage—rarely; every day; and very often (hereinafter referred to as a “frequency-predictor”). Factors such as gender, age, non-verbal fluid intelligence (Raven test), and parental education were considered covariates. The survey of parents showed that more than 93.8% of them had received a university education. Therefore, the parental education covariate was not considered in the final models (the sample for this criterion was almost homogeneous).

To determine whether it is necessary to include the “sex × type of DD usage” interaction factor in the models, we examined the relationship between these factors using the chi-square test. As a result, in all the test groups, neither the boys nor the girls demonstrated any significant preference for the type of DD usage. Therefore, we decided not to include this interaction factor in the final models. All necessary statistical assumptions for constructing multiple linear regressions were successfully tested.

Having constructed all the regression models, we performed power analysis for each of them considering the achieved effect sizes as f_2_ in each model, the size of sample, and α = 0.05 (total 16 power values). Individual characteristics showed statistical significance in some models (see Tables 3–6 for more details). We now proceed to the findings concerning the main type- and frequency-predictors, which were yielded from model simulations carried out for all EF skills and all test groups.

**Table 3:** Multiple linear regression analysis for the study variables predicting visual working memory from the senior kindergarten group to third school grade.

**Model coefficients**	**Model 1**			**Model 2**			**Model 3**			**Model 4**		
	**5–6 y.o**.			**6–7 y.o**.			**7–8 y.o**.			**9–10 y.o**.		
	β	***SE***	***p***	β	***SE***	***p***	β	***SE***	***p***	β	***SE***	***p***
Intercept	60.03	33.1	0.073	46.32	28.93	0.110	182.8	46.22	0.000	67.72	97.83	0.492
Sex	1.398	4.12	0.735	0.98	2.77	0.724	−1.518	4.253	0.722	−3.56	5.628	0.530
Age	0.23	0.50	0.644	0.37	0.37	0.311	−0.918	0.490	0.063	0.470	0.848	0.582
Raven	0.77	0.30	**0.012**	0.34	0.203	0.092	0.757	0.261	**0.004**	0.592	0.423	0.169
Type DD usage	−5.61	3.01	0.065	2.35	1.99	0.239	2.287	2.449	0.351	−2.615	3.638	0.476
Frequency DD usage	−0.998	1.47	0.497	−0.73	2.33	0.756	−0.744	2.102	0.723	1.213	2.932	0.681
*F-statistic (p-value)*	*F*_(6.93)_ = 2.908, *p* = 0.017			*F*_(5.284)_ = 1.255, *p* = 0.283			*F*_(5.164)_ = 2.575, *p* = 0.028			*F*_(5.46)_ = 0.94, *p* = 0.4611		
*Multiple R^2^ (AdjR^2^)*	0.134 (0.09)			0.022 (0.004)			0.0728 (0.045)			0.093 (−0.005)		
*Is the sample size sufficient? (Power-analysis)*	Yes			No			Yes			No		

**Bold** text indicates a statistically significant predictor (*p*-value < 0.05).

**Table 4:** Multiple linear regression analysis for the study variables predicting verbal working memory from the senior kindergarten group to third school grade.

**Model coefficients**	**Model 1**			**Model 2**			**Model 3**			**Model 4**		
	**5–6 y. o**.			**6–7 y. o**.			**7–8 y.o**.			**9–10 y.o**.		
	β	***SE***	***p***	β	***SE***	***p***	β	***SE***	***p***	β	***SE***	***p***
Intercept	10.29	7.81	0.190	1.276	5.58	0.819	10.83	4.181	**0.010**	18.25	21.01	0.389
Sex	1.770	0.95	0.066	1.829	0.54	**0.000**	1.256	0.612	**0.042**	0.001	1.21	0.99
Age	0.113	0.12	0.342	0.231	0.07	**0.001**	0.087	0.043	**0.042**	0.352	0.18	0.059
Raven	0.148	0.07	**0.033**	0.074	0.04	0.061	0.103	0.038	**0.007**	0.032	0.09	0.722
Type DD usage	−0.85	0.70	0.233	0.273	0.39	0.479	0.500	0.358	0.163	1.034	0.78	0.192
Frequency DD usage	−0.73	0.34	**0.034**	−0.254	0.45	0.574	−0.644	0.301	**0.034**	0.361	0.63	0.569
*F-statistic (p-value)*	*F*_(5.101)_ = 4.127, *p* = 0.0018			*F*_(5.287)_ = 5.603, *p* < 0.000			*F*_(5.175)_ = 4.358, *p* = 0.0009			*F*_(5.46)_ = 2.199, *p* = 0.070		
*Multiple R^2^ (AdjR^2^)*	0.134 (0.09)			0.089 (0.073)			0.110 (0.085)			0.192 (0.105)		
*Is the sample size sufficient? (Power-analysis)*	Yes			Yes			Yes			Yes		

**Bold** text indicates a statistically significant predictor (*p*-value < 0.05).

**Table 5:** Multiple linear regression analysis for the study variables predicting inhibition from the senior kindergarten group to third school grade.

**Model coefficients**	**Model 1**			**Model 2**			**Model 3**			**Model 4**		
	**5–6 y. o**.			**6–7 y. o**.			**7–8 y.o**.			**9–10 y.o**.		
	β	***SE***	***p***	β	***SE***	***p***	β	***SE***	***p***	β	***SE***	***p***
Intercept	10.20	0.525	0.000	11.46	3.78	0.002	13.67	3.19	0.000	29.82	15.8	0.065
Sex	0.046	0.064	0.469	0.479	0.36	0.184	0.210	0.46	0.653	−0.52	0.91	0.566
Age	−0.017	0.008	**0.027**	−0.021	0.05	0.658	−0.027	0.03	0.408	−0.15	0.14	0.289
Raven	0.001	0.005	0.857	0.102	0.03	**0.000**	0.113	0.02	**0.000**	0.05	0.07	0.503
Type DD usage	0.003	0.047	0.933	−0.101	0.26	0.695	−0.104	0.27	0.704	1.18	0.59	**0.049**
Frequency DD usage	−0.017	0.023	0.456	−0.166	0.30	0.582	−0.644	0.23	**0.005**	−0.05	0.47	0.904
*F-statistic (p-value)*	*F*_(5.98)_ = 1.28, *p* = 0.2789			*F*_(5.283)_ = 3.45, *p* = 0.0048			*F*_(5.174)_ = 4.922, *p* = 0.0003			*F*_(5.46)_ = 1.338, *p* = 0.265		
*Multiple R^2^ (AdjR^2^)*	0.061 (0.013)			0.057 (0.040)			0.1239 (0.098)			0.126 (0.032)		
*Is the sample size sufficient? (Power-analysis)*	No			Yes			Yes			Yes		

**Bold** text indicates a statistically significant predictor (*p*-value < 0.05).

**Table 6:** Multiple linear regression analysis for the study variables predicting cognitive flexibility from the senior kindergarten group to third school grade.

**Model coefficients**	**Model 1**			**Model 2**			**Model 3**			**Model 4**		
	**5–6 y. o**.			**6–7 y. o**.			**7–8 y.o**.			**9–10 y.o**.		
	β	***SE***	***p***	β	***SE***	***p***	β	***SE***	***p***	β	***SE***	***p***
Intercept	10.66	4.48	0.019	12.60	3.22	0.000	8.650	3.53	0.015	27.43	19.1	0.159
Sex	1.914	0.55	**0.000**	0.334	0.31	0.278	0.019	0.52	0.970	−1.56	1.1	0.164
Age	0.051	0.07	0.455	0.085	0.04	**0.039**	0.039	0.04	0.273	−0.11	0.17	0.504
Raven	0.093	0.04	**0.02**	0.084	0.02	**0.000**	0.051	0.03	0.111	0.02	0.08	0.837
Type DD usage	0.507	0.40	0.212	0.189	0.22	0.393	−0.289	0.30	0.344	0.89	0.71	0.216
Frequency DD usage	0.135	0.20	0.494	0.010	0.26	0.968	−1.045	0.25	**0.000**	−0.14	0.57	0.808
*F-statistic (p-value)*	*F*_(5.101)_ = 3.965, *p* = 0.002524			*F*_(5.286)_ = 4.567, *p* = 0.0005044			*F*_(5.174)_ = 4.223, *p* = 0.001199			*F*_(5.46)_ = 0.8103, *p* = 0.5484		
*Multiple R^2^ (AdjR^2^)*	0.164 (0.123)			0.074 (0.058)			0.1082 (0.082)			0.080 (−0.018)		
*Is the sample size sufficient? (Power-analysis)*	Yes			Yes			Yes			Yes		

**Bold** text indicates a statistically significant predictor (*p*-value < 0.05).

##### 3.2.1.1. Visual working memory

First, for visual working memory, we derived that the type- and frequency-predictors are not significant in any of the test groups (see Table 3). *A posteriori* power analysis showed that the sample size was insufficient for drawing reliable conclusions for Model 2 (visual working memory of 6–7 year-olds) and Model 4 (9–10 year-olds).

##### 3.2.1.2. Verbal working memory

Second, we constructed four equivalent regression models for verbal working memory (see Table 4). As a result, in none of the test groups, the type-predictor appeared to be significant. On the other hand, the frequency-predictor turned out to be significant in Model 1 [*t*_(101)_ = −2.146, *p* = 0.034, *d* = 0.258, 95% CI [−1.42, −0.056]] and Model 3 [*t*_(225)_ = −2.138, *p* = 0.034, *d* = 0.596, 95% CI [−1.23, −0.049]]. The higher the frequency, the lower the score for verbal working memory. *A posteriori* power analysis for all models revealed that the statistical power is sufficient in all cases (minimum power value = 0.992), and the sample size is sufficient everywhere.

##### 3.2.1.3. Inhibition

Third, the equivalent models for inhibition were constructed (see Table 5). The type-predictor was found to be statistically significant only for Model 4 [*t*_(66)_ = 2.02, *p* = 0.0493, *d* = 0.185, 95% CI [0.003, 2.365]]. Children from the “means” group had an inhibition score higher than those who used DD as entertainment. The frequency-predictor showed statistical significance for Model 3 [*t*_(225)_ = −2.801, *p* = 0.005, *d* = 0.159, 95% CI [−1.098, −0.190]]. The higher the frequency, the lower the score for inhibition. *A posteriori* power analysis revealed that the power and sample size were sufficient for all models, except Model 1.

##### 3.2.1.4. Cognitive flexibility

Finally, the equivalent models for cognitive flexibility were constructed (see Table 6). The type-predictor was not significant in any of the test groups. The frequency-predictor revealed statistical significance in Model 3 [*t*_(225)_ = −4.106, *p* < 0.000, *d* = 0.306, 95% CI [−1.548, −0.543]]. The more often a child uses a DD, the lower his cognitive flexibility is. *A posteriori* power analysis revealed that, for all models, the power and sample size were sufficient.

## 4. Discussion

The development of voluntary behavior is considered by most researchers to be a key acquisition of preschool age in the framework of mental development and the child's readiness for school (Vygotsky, 1984; Elkonin, 2006; Smirnova, 2015). However, the features of the development of voluntariness in the era of digitalization are still not so clear (Veraksa et al., 2022). This explains the relevance of the study of voluntariness in the context of digital device usage by modern children. The purpose of this study was to gain a better understanding of the possible factors that are worth considering when studying the impact of digital devices on child development of voluntary behavior. The current study explored the development of voluntary behavior through Miyake's concept of EF skills (Miyake et al., 2000). Within the framework of research on this topic, the most widely studied factor is screen time. However, children spend more and more time with gadgets, but not in all cases are negative effects on development observed. It is worth refocusing on other factors since the factor of screen time has limitations and cannot always explain the details of the influence of digital devices on child development (Christakis et al., 2013; Huber et al., 2016; Radesky and Christakis, 2016; Papadakis et al., 2022; Veraksa et al., 2022). In the current study, we suggest DD usage as a factor for studying the impact of digital devices on child EF. This factor was considered in terms of the cultural–historical activity perspective as one of the psychological means that mediate human activities and communication. This view on DD has been already proven to be admissible by other authors working within the cultural–historical perspective (Vojskunsky, 2005; Nechaev and Durneva, 2016; Rubtsova, 2019a,b).

The findings of the present study show that a change in the type of DD usage takes place as children grow older. In kindergarten, most participants use DDs as entertainment, which agrees with results in other studies (Nechaev and Durneva, 2016; Papadakis et al., 2018; Gözüm and Kandir, 2021; Nikolaeva and Isachenkova, 2022; Veraksa et al., 2022). By the third grade, the number of children who use the DD as a psychological means increases. This corresponds to the change of leading activity from playing to learning from preschool to primary school age (Elkonin, 1999; Sivrikova et al., 2020; Zain et al., 2022). The main hypothesis of the present study was that DD usage as a psychological means is associated with a higher EF skill level than when DDs are used for entertainment. Sixteen multiple regression models were constructed separately for each of the four EF skills and for all four groups under study. In the models, the “type of DD usage” (as a means or for entertainment) and “frequency of DD usage” (rarely, every day, and very often) were used as predictors. The individual characteristics of the participants were also considered covariates. As a result, only one model for inhibition found statistical significance for the “type of DD usage.” Consistent with this fact, the 9–10-year-old children using gadgets as a means have inhibition scores higher than those who use DD as entertainment. In any other case, this factor was not significant. The hypothesis was not confirmed. When it comes to the frequency of DD usage (an equivalent of the screen time factor), this factor was significant in four cases. The more often children aged 7–8 years use digital devices, the worse their verbal working memory, inhibition, and cognitive flexibility develop. Furthermore, the more often the children 6–7 years old use gadgets, the worse their verbal working memory develops. Thus, the factor of frequency of DD usage can appear to be quite productive for the study of executive functions in the context of children's digital device exposure. However, it is worth paying attention to the trend toward statistical significance for the differences between average values of EF skills. It is interesting to note that, in 10 out of 16 cases (four EF skills across four test groups), the average values of EF skills for the “means” group exceeded those for the “entertainment” group. However, at the age of 6–7 years, the children using gadgets as a means have higher scores in the executive functions of visual working memory, verbal working memory, inhibition, and cognitive flexibility. The children of 7–8 years also had higher scores in both visual and verbal working memory. The children aged 9–10 years also demonstrated higher scores across all executive functions. Such a tendency was not found for children aged 5–6 years.

Even though only in three out of sixteen cases the “means” group showed significantly higher results in executive functions, the presence of the trend toward statistical significance for differences between average values of EF skills is still important. This pattern of results is consistent with that of the previous research (Veraksa et al., 2022). It is also consistent with the theoretical assumption that the means modify the structure and features of the mental function's activity (Luria, 1928; Vygotsky, 1982).

The results obtained can be analyzed from the point of view of cultural–historical perspective as follows. For example, it has been shown that children aged 9–10 years using DD as a means have better inhibition scores (according to regression models considering individual characteristics of participants). Inhibition was assessed using a task where the child needed to name the figures oppositely to what the child really sees. In other words, the child had to switch from the physical plane to the mental one. Thus, while performing the inhibition task, the child acts in accordance with the internal mental plan and not under the influence of a motive coming from a physical object (he calls the square a circle, although the image of the square seems to motivate the child to say “square”). Consequently, the children who cope with this task well have a better ability to switch from the material plane to the mental one, and it is voluntariness, according to the concept of Vygotsky (1966). At the same time, the studies showed that many digital games do not include shifting to the mental plane but make the child act mainly in accordance with the material one (Veraksa et al., 2022). This probably leads to the fact that children who spend a lot of time playing digital games have little experience in shifting to the mental plane (which can be acquired during role-playing games, communication with others, etc.). The role-playing game combines necessary conditions for the development of voluntary behavior—increasing motivation and awareness of one's behavior. Elkonin noted that the rule is incorporated into the role during the game and the child monitors and regulates his behavior to comply with this rule (Elkonin, 2006). Thus, the participation in a game situation is a springboard for the development of voluntary behavior. At the same time, communication allows the child to gain experience with speech signs' usage. According to Vygotsky, the use of speech signs is the most important means of mediation (Vygotsky, 1983). Thus, children who interact with gadgets very often have less experience in live communication and participation in role-playing games, which increases the risk of incomplete voluntary behavior development. The opposite is also true: children who communicate more often and take part more often in role-playing games develop the ability to voluntarily switch to the mental plane more effectively. There is a dual affective plan in a role-playing game: the child experiences the pleasure of restraining direct impulses, subordinating his behavior to the meaning of what is happening in the game situation and to the role taken (Vygotsky, 1966). The restraining of impulses is inhibitory control.

In our view, there are three compelling explanations for our findings. First, the negative result (insignificance of differences between groups and therefore, the insignificance of the “type of DD usage” factor in most regression models) may have resulted from a lack of statistical power—type II error. The power analysis comparing the “means” group and the “entertainment” group showed a maximum power of 68% in all 16 comparisons, which supports the aforementioned assumption. It is not enough to completely reject the hypothesis that there are differences between the children who displayed different types of DD usage (Cohen, 1962). The sample size of the “means” group was not large enough to show a significant effect according to the results of power analysis. The size of the “entertainment” group was satisfactory. The point is that it is difficult to recruit a considerable number of children who use gadgets mainly as a psychological means to regulate their own or someone's else behavior but not mainly for entertainment. A more typical pattern of activity for children of this age is entertainment. Second, in addition to the insufficient sample size, a negative result may arise from the interview design. Factors such as “type” and “frequency” of DD usage were obtained according to the parent's reports (for the children of 5–6 years of age) and according to the child's reports (for the 6–7, 7–8, and 9–10-year-olds). Thus, a discrepancy may exist between the reported and the actual DD usage. The subjective assessment of screen time and type of digital activity can often differ from the actual digital activity of children (Gentile et al., 2012; Nikitina and Rytova, 2019). Third, we cautiously assumed that it is better to use the type-predictor for older ages than for the younger ages corresponding to kindergarten and early primary school. This predictor is not so predictive for studying the characteristics of young children's development. That is because DD usage for anything other than entertainment generally does not apply to the lives and interests of young children (according to the results, up to 60% of the kindergarten children use DD for entertainment). Play activity is the leading type of activity at this age (Elkonin, 1999). This is confirmed by the lower number of children using the DD as a means in kindergarten in our study (up to 10%). However, we assumed that the type-predictor will be significant for older ages at the end of primary school and beyond. In our study, this factor turned out to be significant only in the oldest group of 9–10-year-olds (closer to the end of primary school). The representative number of children using DD as a means should already be large enough by this age, due to a fundamental change in the children's activities from playing to learning and to the constantly increasing need for self-regulation of daily routine in order to become less dependent on parents (Sarsekeyeva et al., 2016). Indeed, this study demonstrated that, the number of children using DD as a means significantly increases with age (7.4% for the 5–6-year-old children and 38% for the 9–10-year-old children). We assume that children older than 9–10 years of age use DD as a means more often than those who are younger, and therefore, the type-predictor will be more appropriate for this age group. For example, when it comes to preschoolers, playing with social roles or digital games organized in the format of an online game with social roles should be organized in the preschooler's leisure time (Solovieva and Quintanar, 2021). Playing with social roles is important for preschool development since this is the leading activity at this age (Elkonin, 1999). Therefore, it is important not only to assess the fact of using digital devices but also to control the features of normative child development. The present study considered the children's intellectual development (Raven test) and the parental educational level. However, it may be worth monitoring other aspects that determine the normative development of a child of this age period as far as possible.

The trend toward the statistical significance of differences between mean values for some EF skills was displayed. Thus, the children using DD as a means showed higher EF skills. Despite the lack of significance in the analyzed cases, the presence of the aforementioned trend might prove the application of the cultural–historical perspective to the research on child development in the digital era when DDs have become an important psychological means for self-regulation. Hence, it is crucial to determine the age interval as to when the type-predictor might be most applicable. This proves the actuality of the results of our study. It must be considered that the contemporary digital era is quite different from pre-digital times when the cultural–historical perspective evolved. First, DD usage as a psychological means entails a partial transition to a new type of externalization. In particular, the actions, which used to be completely internalized before, have partially moved to the DDs' area of responsibility (Falikman, 2021). Second, digital devices supplant playing with social roles more and more, while their developmental potential is still considered crucial for the preschool age in terms of the cultural–historical perspective of the pre-digital era (Smirnova and Sokolova, 2013; Hakkarainen and Bredikyte, 2020; Zulkifli et al., 2021; Yudina, 2022). Considering all the aforementioned factors, we suggest that the application of the cultural–historical perspective elaborated during the pre-digital era requires to be revised in contemporary digital times.

## 5. Limitations

The current study is based on data from children's and parent's reports on digital device usage, which could result in inaccuracy in the assessment of children's digital activity. Furthermore, a longitudinal design is required to draw reliable conclusions about the relationship between voluntariness and digital device usage. Despite these limitations, the results suggest some theoretical and practical implications.

## 6. Conclusion

In the current study, the type of DD usage as a psychological means or as entertainment was suggested to be considered as a factor for studying the impact of digital devices on child voluntariness. The voluntary behavior was evaluated using the level of executive functioning development. The hypothesis under study was that DD usage as a psychological means is associated with higher executive functioning scores acquired by children from kindergarten (5–6 years old) to mid-primary school (9–10 years old) age. “The type of digital devices usage” (as a psychological means or for entertainment) and “the frequency of digital devices usage” predictors were tested. The “frequency of digital device usage” predictor proved its statistical significance for verbal working memory, inhibition, and cognitive flexibility at 7–8 years, and for verbal working memory at 6–7 years. The higher the frequency, the lower the score for these executive functions. The type-predictor proved to be significant only for inhibition at 9–10 years. Children with DD usage as a means show an inhibition score higher than those who use DD as entertainment. However, type-predictor revealed no significance for other executive functions. We explained this result as follows: it is not productive to attribute the DD activity of a child of 5–10 years of age to one of the two categories—as a psychological means or for entertainment—because of the very small percentage of children who use gadgets as a means at this age. The number of children using DDs as a means increases significantly by the end of primary school. This is a consequence of the leading type of child activity changing from playing to learning, as well as to the increasing need of the child to engage in voluntary behavior without parental control. The type-predictor is thought to be more applicable in the studies of the voluntariness–DD exposure relationship involving children in the final grade of primary school and older. We hope that the current study will stimulate further investigation in this important research area.

## Data availability statement

The raw data supporting the conclusions of this article will be made available by the authors, without undue reservation.

## Ethics statement

The studies involving human participants were reviewed and approved by Ethics Committee of the Faculty of Psychology at Lomonosov Moscow State University (approval no: 2018/54). Written informed consent to participate in this study was provided by the participants' legal guardian/next of kin.

## Author contributions

AS conceived, conceptualized, designed the study, gathered, and analyzed the data. MG acquired resources. AS, EC, and MG drafted the manuscript. All authors contributed to the article and approved the submitted version.
